# Is Correction of Iron Deficiency a New Addition to the Treatment of the Heart Failure?

**DOI:** 10.3390/ijms160614056

**Published:** 2015-06-18

**Authors:** Donald S. Silverberg, Dov Wexler, Doron Schwartz

**Affiliations:** 1 Department of Nephrology, Tel Aviv Medical Center, Tel Aviv 64239, Israel; E-Mail: doron@tasmc.health.gov.il; 2 Department of Cardiology, Tel Aviv Medical Center, Tel Aviv 64239, Israel; E-Mail: wexlerd@bezeqint.net

**Keywords:** anemia, iron, iron deficiency, intravenous iron, erythropoietin, heart failure, renal failure

## Abstract

Anemia is present in about 40% of heart failure (HF) patients. Iron deficiency (ID) is present in about 60% of the patients with anemia (about 24% of all HF patients) and in about 40% of patients without anemia (about 24% of all HF patients). Thus ID is present in about half the patients with HF. The ID in HF is associated with reduced iron stores in the bone marrow and the heart. ID is an independent risk factor for severity and worsening of the HF. Correction of ID with intravenous (IV) iron usually corrects both the anemia and the ID. Currently used IV iron preparations are very safe and effective in treating the ID in HF whereas little information is available on the effectiveness of oral iron. In HF IV iron correction of ID is associated with improvement in functional status, exercise capacity, quality of life and, in some studies, improvement in rate of hospitalization for HF, cardiac structure and function, and renal function. Large long-term adequately-controlled intervention studies are needed to clarify the effect of IV iron in HF. Several heart associations suggest that ID should be routinely sought for in all HF patients and corrected if present. In this paper we present our approach to diagnosis and treatment of iron deficiency in heart failure.

## 1. Introduction

The role of iron deficiency (ID) in heart failure (HF) has been the subject of many recent reviews over the last 2 years [1,2,3,4,5,6,7,8]. About 40% of patients with HF have anemia as defined as a hemoglobin (Hb) of less than 13 g% in men and 12 g% in women, and 60% do not have anemia [1,2,3,4,5,6,7,8]. When ID in HF is defined as either a serum ferritin of <100 µg/L or a serum ferritin of 100–300 µg/L along with a percentage of % transferrin saturation (TSat) (serum iron divided by transferrin levels multiplied by 100-%TSat) of <20%, about 60% of the anemic patients (24% of all HF patients) and about 40% of the non-anemic patients (also 24% of all HF patients) have ID [1,2,3,4,5,6,7,8,9,10,11,12,13,14,15]. Thus, about half the patients seen with HF have ID [9,10,11,12,13,14,15].

## 2. Associations of Heart Failure (HF) and Iron Deficiency (ID)

The presence of ID in HF has been confirmed in studies showing reduced iron content in the bone marrow [16] and in the heart [17,18,19]. The more severe the ID the more common and severe are adverse cardiovascular events [9,10,11,12,13,14,19]. Indeed ID has, independent of anemia, been associated with increased morbidity, hospitalization and mortality in HF [9,10,11,12,13,14,15,19] including lower Quality of Life (QoL) [9,10,11,12,13,14,15,20]. But these studies have only been done with systolic HF and not diastolic HF.

## 3. Effect of Intravenous (IV) Iron Treatment of HF

But these associations between morbidity and mortality in HF and ID may not be causal. To prove that ID is actually causing damage one must treat the ID and see the response of the HF patient. Although no long term studies have been published that have shown that treatment of ID can improve HF, five placebo-controlled randomized studies using intravenous (IV) iron [21,22,23,24,25] lasting up to 12 months have been published as well as three uncontrolled studies [26,27,28] (Table 1).

In these studies the treatment with IV iron has been shown to improve the anemia [21,22,23,24,25,26,27,28], the New York Heart Association (NYHA) functional class [21,22,23,24,25,26,27,28], the six minute walk distance [21,22,26,27,28], oxygen utilization during exercise [22] and Quality of Life (QoL), as judged by several different parameters [21,22,23,26,27,28,29], and has indeed been shown to be one of the most cost-effective interventions for improving QoL available in HF [30,31,32,33,34].

The QoL parameters that are improved include reduced physical limitation, improved mobility, improved self care, increased daily activities, and reduced general pain, discomfort, anxiety and depression [23,29].

The IV iron treatment also improved renal function in several studies [21,23,25,35]. The cause of this improvement in renal function is unknown but is possibly related to the improvement in cardiac function.

ID correction also reduced several prognostic markers associated with worsening of HF including levels of C-reactive protein [21,25], beta natriuretic peptide [21,25] and red cell distribution width (RDW) [36].

**Table 1:** A summary of 5 controlled studies and 3 uncontrolled studies on the use of intravenous iron in heart failure.

Authors and Reference	Controlled or Uncontrolled Study	Number of Patients	Iron Treatment	Duration of Follow-up	Study Results
Bolger *et al.* [26]	Uncontrolled	16	Iron sucrose	92 days	Improvement in NYHA, MLHF questionnaire score and 6MWD
Usmanov *et al.* [27]	Uncontrolled	32	Iron sucrose	26 weeks	Improvement in cardiac remodeling: PWT ↓, LVEDD↓, LVEDV↓, LVESD↓, LVESV↓, LVMI↓; LVEF increased and NYHA improved in NYHA III
Gaber *et al.* [28]	Uncontrolled	40	Iron dextran	12 weeks	Improved NYHA and 6MWD. The S', E' wave values and E/E' ratio values improved. Peak systolic strain rate showed marked improvement—all the above are signs of improved myocardial function
Toblli *et al.* [21]	Controlled	40	Iron sucrose	6 months	Reduction in NT-proBNP, CRP. Improved LVEF, NYHA, exercise capacity, renal function and QoL and fewer hospitalizations
Okonko *et al.* [22]	Controlled	35	Iron sucrose	18 weeks	Increase in peakVO_2_/kg, improved NYHA, and patient global assessment
Anker *et al.* and others [23,29,31,35,36]	Controlled	459	Ferric carboxymaltose	24 weeks	Improvement in NYHA, patient global assessment, 6MWD, renal function, RDW, Quality of Life and patient global assessment, compared to control group Trend to decreased CV hospitalizations. Improved cost effectiveness
Ponikowski *et al.* [24]	Controlled	304	Ferric carboxymaltose	52 weeks	Improvements in Patient Global Assessment, NYHA, QoL, 6MWD, Fatigue score, Reduced risk of hospitalization for worsening heart failure
Toblii *et al.* [25]	Controlled	60	Iron sucrose	6 months	Improved NYHA. Reduction in CRP and NT-Pro BNP. LVSD↓, LVDD↓, LVPWT↓; Improved LVEF and renal function

CRP: C Reactive Protein; NT pro BNP: N Terminal pro Natriuretic Peptide; LVSD: Left Ventricular Systolic Diameter; PWT: Posterior Wall Thickness; LVEDD: Left Ventricular End Diastolic Diameter; LVEDV: Left Ventricular End Diastolic Volume; LVESD: Left Ventricular End Systolic Diameter; LVESV: Left Ventricular End Systolic Volume; LVMI: Left Ventricular Mass Index; LVEF: Left Ventricular Ejection Fraction; MLHF: Minnesota Living With Heart Failure Questionnaire; NYHA: New York Heart Association; QoL: Quality of Life; RDW: Red Cell Distribution Width; 6MWD: 6 Minute Walking Distance.

The correction of ID also reduced admissions for HF in some studies [21,24]. When the results of four of the five placebo-controlled studies were pooled [21,22,23,24] there was a significant 67% reduction in hospitalization for HF compared to the placebo-controls [8]. IV iron can also improve cardiac dysfunction as measured by tissue strain using a Doppler method [28], reduce left ventricular hypertrophy and dilatation [25,27] and increase left ventricular ejection fraction (LVEF) [25,27].

Although the anemia is also improved with the IV iron therapy, the improvements in HF have been shown to be independent of the improvement in anemia [21,22,23,24,25]. But it is still uncertain how much of the cardiac improvement with IV iron is due to the correction of the anemia and how much is due to the improvement in the ID since both Hb and iron are important in improving cellular function and cardiac function.

## 4. Official Recommendations for the Diagnosis and Treatment of Iron Deficiency in HF

The European [37] and the Australia-New Zealand [38] Societies of Cardiology, and a French expert cardiac committee [39] now recommend including an iron evaluation as part of the routine work-up of HF and treating the ID whenever it is discovered. In contrast the recently published American Society of Cardiology guidelines for HF state that “In the absence of a definitive evidence base, the writing committee has deferred a specific treatment recommendation regarding anemia until ongoing randomized trials are completed” [40]. Similarly the American College of Physicians [41] and the Canadian Cardiovascular Society [42] make no specific recommendations about routine iron investigation in HF.

Despite such guidelines we recently found that only 19.7% of patients admitted to our hospital with a primary diagnosis of HF had their iron parameters measured, yet in those in whom it was measured, 80% were found to be iron deficient [43,44]. A similar lack of investigation and treatment of ID in HF has been found by others [45,46].

## 5. Causes of Iron Deficiency

There are many causes of ID in Renal Failure (RF) and HF [1,2,3,4,5,6,7], including:

(1) Reduced iron intake due to low protein diets and anorexia;
(2) Gastrointestinal blood loss due to uremia, gastritis, tumors, platelet inhibitors, anticoagulants and phosphate binders (which can also bind iron). In addition HF itself can cause reduced iron absorption due to intestinal cell dysfunction due to bowel edema and other causes;
(3) Erythropoietin (EPO) used for the treatment of anemia uses up iron to form Hb, and this eventually will cause ID;
(4) Inflammation.

Both RF and HF are inflammatory conditions cause increased cytokines, and this can cause anemia and ID. These cytokines include Tumor Necrosis Factor-α (TNFα) and interleukin-6 (IL-6). They can cause four hematological abnormalities:

(4.1) Reduced EPO production in the kidney leading to inappropriately low EPO levels in the blood for the degree of anemia present;
(4.2) Reduced erythropoietic response of the bone marrow to Erythropoiesis Stimulating agents (ESA);
(4.3) Hepcidin-induced failure of iron absorption from the gut;
(4.4) Hepcidin-induced trapping of iron in iron stores in the macrophages and hepatocytes preventing the release of iron into the blood.

The latter two abnormalities listed above have been reported to cause ID.

Hepcidin is a protein released from the liver by IL-6. It inhibits the protein ferroportin, which is found in the gastrointestinal tract and in macrophages and hepatocytes and is responsible for the release of iron from these three types of cells into the blood. Therefore, if ferroportin is inhibited, gastrointestinal iron absorption is diminished, and iron also is not released from its storage in macrophages and hepatocytes. This results in decreased levels of iron in the blood which leads to decreased delivery of iron to the bone marrow and, therefore, ID anemia, even in the presence of adequate total iron stores (this is the so-called functional iron deficiency or the anemia of chronic disease). Because hepcidin is filtered and removed in the kidney, its levels increase even further in Renal Failure (RF) which can also partly explain the ID in this condition [47]. About half of all patients with HF have renal insufficiency as judged by a creatinine clearance of less than 60 mL/min/1.73/sqm [1,2,3,4,5,6,7,8,9,10,11,12,13,14,15].

## 6. What Are the Cellular Effects of Iron Deficiency?

There are hundreds of enzymes in the body that depend on iron for their function [48]. Iron is indispensable for life, playing a crucial role in oxygen transport (through the production of hemoglobin in the red cell), oxygen storage and diffusion in the cell (myoglobin), and as a component of oxidative enzymes and respiratory chain proteins involved in energy production in the mitochondria in all cells. Iron is also involved in the synthesis and degradation of proteins, lipids, catecholamines, collagen, ribonucleic acids and DNA.

## 7. Iron Toxicity and Safety

Despite the positive studies of IV iron in HF and RF there are no long-term controlled studies that have been done on the use of IV iron in HF patients and there is concern about the safety of such preparations [49,50] Excessive iron may cause increase oxidative stress, endothelial injury and dysfunction, atherosclerosis and cardiovascular disease. It can cause altered immunity with T-Cell lymphocyte dysfunction and impaired phagocytic function of polymorphonuclear cells which can cause increased susceptibility to infections. Excessive iron can also cause DNA damage and mitochondrial membrane instability. Clearly more long-term adequately-powered and placebo-controlled studies are required on IV iron in HF to evaluate the safety of IV preparations. However the studies done up to now show that these agents appear to be very safe with the exception of High Molecular Weight Dextran which appears to have a relatively high incidence of Dextran-related anaphylaxis side effects [51,52,53,54,55,56,57,58]. All the other IV iron agents; Ferric carboxymaltose, ferric sucrose, ferric gluconate, ferumoxytal and iron maltoside 1000 all seem to have a very low incidence of side effects and are very effective in the treatment of ID [51,52,53,54,55,56,57,58]. Low molecular weight dextran is also available and has a lower incidence of dextran-related side effects than the high molecular weight dextran but the fact that it contains dextran is still a cause for concern [7]. The rate of administration is different for each of these compounds. Ferric sucrose and ferric gluconate have to be given in lower doses so that they need to be given more frequently than the other compounds which can be given in higher doses and therefore less frequently. The total dose given initially is usually about 1000 mg over one to five injections over a few weeks and subsequent administration depends on the response to therapy [51,52,53,54,55,56,57,58]. All the five controlled studies of IV iron in HF mentioned above were performed with either ferric sucrose or ferric carboxymaltose (Table 1).

## 8. Animal Studies

Iron deficiency in animal studies has shown the development of left ventricular hypertrophy and dilatation, mitochondrial swelling, disruption of sarcomeres and release of reactive oxygen species that can cause cell damage [59].

## 9. Oral *versus* Intravenous Iron in Chronic Kidney Disease and Congestive Heart Failure

In most studies comparing oral to IV iron in RF, IV iron has been found to produce a greater and more rapid Hb response than oral iron with fewer side effects, and many patients can reach a target Hb of 11.0–11.5 g/dL with IV therapy alone, and therefore avoid the use of Erythropoiesis Stimulating Agents (ESA) altogether [58,60]. In addition the serum ferritin and %TSat increase more and more rapidly with the iron therapy [58,60]. The IV iron avoids the poor absorption, gastrointestinal symptoms, and noncompliance that frequently occurs with oral preparations [58,60]. Very little information exists about the use of oral iron in ID in HF. Some studies suggest that oral iron had little effect on the Hb levels, iron parameters or any cardiac parameters [7]. However in one trial of HF, 30 patients received oral iron and 30 did not [61]. The scores for Hb and RBC parameters improved only in the oral iron group and QoL, dyspnea and fatigue all improved in both groups but more in the oral iron group. In another small controlled study [62] oral and IV iron had similar increases in Hb but there was a significant increase in peak oxygen utilization (VO_2_) only in the IV iron group. However the study was underpowered with only 18 patients completing it. In a retrospective study of 105 patients with HF with systolic dysfunction oral iron supplementation improved iron stores and anemia similarly to previously reported results with intravenous iron therapy suggesting that oral iron therapy may be useful. But, as stated this study was retrospective [63]. Much more data is therefore needed on oral agents in HF. In patients with cyanotic congenital heart disease who were polycythemic and had ID, oral iron improved the Hb, the QoL and the 6 Minute Walking Distance as well as serum ferritin and %TSat, but not peak VO_2_ [64] This uncertainty about which treatment to use initially, oral or IV iron, is reflected in expert reviews in HF, some of which recommend starting with oral iron [5] and some with IV iron [7]. Clearly if the patient feels well and his condition is stable there may be no harm in starting with the oral iron but in symptomatic or unstable patients, where time is of the essence, it would seem reasonable to begin with IV iron treatment.

## 10. Cardiac Response to Iron Deficiency Anemia in Patients without Known Heart Disease and the Effect of Its’ Correction with Oral Iron

In patients with ID who had anemia without known heart disease and arrythmias and who were treated with oral iron until the anemia was corrected, echocardiography was performed before and after treatment. At baseline there was an increase in Left Ventricular Diameter (LVD), Left Ventricular Mass index (LVMI), Left Atrial Volume Index (LAVI), and increased LV filling pressure. The follow up results, similar to the findings in the HF studies mentioned above, showed that those treated with IV iron had significant decreases in LV end-diastolic and end-systolic diameters and LV Mass index. In addition there was evidence of decreased LV filling pressure [65]. Thus oral iron in ID patients without underlying heart and renal disease can successfully correct the ID and cardiac abnormalities. But, as mentioned above, whether this would also be the case in HF has yet to be proven.

## 11. Other Conditions in Which Iron Deficiency Has Been Corrected and Found to Improve the Situation in both Anemic and Non-Anemic Patients

ID is one of the most common conditions in the world. It may appear with anemia but more often is present for many years before anemia develops. In addition its correction has been found to improve many other conditions including: chronic kidney disease (CKD) [66,67,68], inflammatory bowel disease (IBD) [69,70], cancer [71,72], athletic disturbances [73,74,75], rheumatoid arthritis [76,77], restless legs syndrome [78,79], aortic stenosis [80], anemic and non-anemic menstruating women [81,82,83], pregnant and post-partum anemic and non-anemic women [84,85,86,87], anemic and non-anemic young children [88], acute myocardial infarction [89], ID-induced thrombocytosis [90,91], idiopathic pulmonary hypertension [92,93,94], and in perioperative patients [95].

## 12. Non-Intervention Studies of Conditions Associated with ID

There are many conditions in which the prevalence of ID is increased but no adequate studies of iron administration have been performed to assess their effect on ID, anemia and patient performance. These include: heart and kidney transplantation [96], obesity [97,98,99], metabolic syndrome [100], diabetes [101], elderly [102], stroke [103], peripheral vascular disease [104], coronary artery disease [105,106,107,108,109], thromboembolism [110], chronic obstructive pulmonary disease (COPD) [111,112] and asthma [113].

## 13. Why Would ID Increase the Risk of Vascular Disease?

One reason for the increased risk of stroke, coronary artery disease, thromboembolism and peripheral vascular disease seen with ID could be the thrombocytosis and increased platelet aggregation caused by the ID [90,91,101].

## 14. Diagnosis and Treatment of ID

To diagnose ID in HF and other conditions one would have to measure the iron parameters serum iron, serum transferrin, %Transferrin Saturation (%TSat), and serum ferritin [114,115,116,117,118] in any patient suspected of having ID and not just measure them in those with anemia.. The two commonly used tests that are used to detect ID are %TSat and serum ferritin. But although they are both low in absolute iron deficiency, where iron stores are low, in functional iron deficiency, which is often seen in inflammatory states such as HF or RF, iron stores may be normal or high, %TSat will be low but serum ferritin may be normal or high, yet the patient may well respond to IV iron because, as mentioned earlier, (a) iron absorption in the gut is low and (b) the iron remains in the iron stores and is unable to be released into the blood and go to the bone marrow for aiding erythropoiesis.

There is no test that can predict very accurately the degree of response to IV iron in HF or RF or in other inflammatory conditions with great accuracy [114,115,116,117,118]. Some suggest that analysis of the soluble transferrin receptor best reflects unmet cellular iron requirements and is the most accurate measurement for predicting the severity of the ID and the response to IV iron [116]. Others suggest the use of various red cell parameters such as the percentage of microcytic erythrocytes or hypochromic erythrocytes, or the level of hemoglobin in reticulocytes and mature red blood cells [117,118]. Others suggest that hepcidin best predicts Hb response to oral iron [119]. More information is needed to answer this question in HF.

## 15. Guidelines for When to Stop the IV Iron

There currently are no generally agreed upon guidelines as to when to start or stop treatment for IV iron in HF. Many studies have recently used either a serum ferritin of <100 µg/L or a serum ferritin of 100–300 µg/L associated with a %TSat <20% in HF. According to the Clinical Renal Guidelines for Iron Deficiency Treatment in Renal Failure [66,67], IV iron therapy can be given to RF patients until either the %TSat reaches 30% or the serum ferritin reaches 500 µg/L. The usual total initial dose as mentioned above, is 1000 mg of elemental iron given IV in one to five sessions over one to several weeks depending on which iron preparation is used. Further iron is given as needed depending on the Hb and iron indices responses. We have used similar guidelines for HF.

## 16. Other Nutritional Deficiencies

Deficiencies in Vitamin B12 and Folic Acid may each be found in about 5% of the HF patients and should be routinely sought for and treated [120].

## 17. Lack of Awareness of the Importance on Non-Anemic Iron Deficiency

Non anemic iron deficiency is very common [121,122]. It may be a major cause of symptoms as shown above but is often missed [43,44,45,46,112,122]. Health personnel are apparently often not aware of the presence and importance of ID without anemia [122] and should be made aware of this very common entity.

## 18. Erythropoiesis Stimulating Agents (ESA)

A large study of Erythropoiesis Stimulating Agents (ESA) in HF has recently been published, the Reduction of Events with Darbepoetin alfa in Heart Failure (RED-HF) study, which was undertaken in 2006 and recently completed [123,124]. There was no significant between-group difference in any of the primary or secondary outcomes. The neutral effect of Darbepoetin alfa was consistent across all prespecified subgroups. Fatal or nonfatal stroke occurred in 42 patients (3.7%) in the Darbepoetin alfa group and patients (2.7%) in the placebo group (*p* = 0.23). Thromboembolic adverse events were reported in 153 patients (13.5%) in the Darbepoetin alfa group and 114 patients (10.0%) in the placebo group (*p* = 0.01). There was no difference in renal function or progression of renal disease in the treated and untreated group. Cancer-related adverse events were similar in the two study group. The overall findings did not support the use of Darbepoetin alfa in these patients. Thus a positive role for ESA in HF has not been proven [124,125]. We feel it should only be used in low doses after the ID has been corrected with IV iron and the patient’s Hb is still below 10 g%.

## 19. Conclusions

Our personal approach based on the current data is

(1) All patients with heart failure should routinely have a serum iron, serum Transferrin, % Transferrin Saturation and serum ferritin performed in addition to the usual blood work irrespective of whether anemia is present or not.
(2) IV Iron therapy can be given to heart failure patients with serum ferritin <100 µg/L or serum ferritin 100–300 µg/L with %TSat <20% until either a %TSat of 30% or a serum ferritin of 500 µg/L has been reached.
(3) Current IV iron preparations are all quite safe and can be used with the exception of High Molecular Weight Dextran which should not be used because of it allergic potential.
(4) If the clinical situation is not acute, unstable, or symptomatic, oral iron can perhaps be used but if the patient is unstable or symptomatic then consideration should be given to a course of IV iron which corrects both the ID and anemia more rapidly, to a greater degree and in a higher percentage of patients than oral iron therapy and more completely corrects the ID.
(5) Erythropoietin injections should only be used once ID has been corrected and the Hb is still <10 g/dL. If erythropoietin agents are used they should only be used in low doses because of the dangers of thromboembolic phenomena and hypertension associated with high doses of ESA.
(6) The target Hb for heart failure is usually accepted to be around 11.5 g/dL.
(7) Treatment of heart failure with IV iron may prevent the progression of the renal disease as well as the heart disease.
(8) Secondary causes of worsening heart failure and renal failure such as Non Steroidal Anti Inflammatory Drugs (NSAIDs), hypotension, urinary retention and excessive salt intake should be routinely looked for.
(9) Preoperative correction of ID with IV iron may prevent transfusions and their associated cardiovascular complications in the postoperative period.
(10) Vitamin B12 and folic acid levels should be measured in all HF patients and deficiencies treated.
(11) The cause of ID should be looked for when ID is found.

## 20. Major Challenges

There are still many uncertainties about whether to begin with IV iron or oral iron in HF: (1) The lack of a long-term double- blind placebo-controlled studies of a size adequate to assess morbidity and mortality is a major problem for both the IV and oral iron treatment in HF; (2) Doctors should be made more aware that ID can be present and cause serious signs and symptoms even when no anemia is present; (3) More data on safety of the IV iron preparations in HF is needed (4) Do hospitalization rate and mortality really improve in HF with IV iron therapy? (5) Does cardiac function really improve in HF with IV iron therapy? (6) Does renal function really improve in HF with the IV iron therapy? (7) Better parameters are needed to assess iron deficiency. Should Hepcidin, transferrin receptor protein, % hypochromic red cells and/or reticulocyte Hb concentration become routine tests in the search for ID? (8) Is there a role for oral iron therapy in the treatment of ID of HF? (9) Does the same IV iron therapy work in diastolic heart failure with a normal ejection fraction as it does in systolic heart failure? (10) Is ID really more common in the elderly, obesity, diabetes, metabolic syndrome, coronary heart disease, acute myocardial infarction, peripheral vascular disease, stroke, thromboembolic diseases, COPD, asthma and rheumatoid arthritis? If so is there a role for correction of ID in these conditions?
